# The Left Ventricular Myocardium in Hypoplastic Left Heart Syndrome

**DOI:** 10.3390/jcdd9080279

**Published:** 2022-08-19

**Authors:** Bill Chaudhry, Ahlam Alqahtani, Lorraine Eley, Louise Coats, Corina Moldovan, Srinivas R. Annavarapu, Deborah J. Henderson

**Affiliations:** 1 Bioscience Institute, Faculty of Medicine, Newcastle University, International Centre for Life, Newcastle upon Tyne NE1 3BZ, UK; 2 Adult Congenital Heart Disease Unit, Freeman Hospital, Newcastle upon Tyne Hospitals NHS Foundation Trust, Newcastle upon Tyne NE7 7DN, UK; 3 Population Health Sciences Institute, Newcastle University, Newcastle upon Tyne NE2 4HH, UK; 4 Department of Cellular Pathology, Royal Victoria Infirmary, Newcastle upon Tyne Hospitals NHS Foundation Trust, Newcastle upon Tyne NE1 4LP, UK; 5 Department of Paediatric Histopathology, Alder Hey Children’s NHS Foundation Trust, Eaton Road, Liverpool L12 2AP, UK

**Keywords:** endocardial fibroelastosis, slit-like ventricle, peach-like ventricle, mitral atresia, mitral stenosis, aortic atresia, aortic stenosis, senescence, myocyte disarray

## Abstract

Hypoplastic left heart syndrome (HLHS) is a collective term applied to severe congenital cardiac malformations, characterised by a combination of abnormalities mainly affecting the left ventricle, associated valves, and ascending aorta. Although in clinical practice HLHS is usually sub-categorised based on the patency of the mitral and aortic (left-sided) valves, it is also possible to comprehensively categorise HLHS into defined sub-groups based on the left ventricular morphology. Here, we discuss the published human-based studies of the ventricular myocardium in HLHS, evaluating whether the available evidence is in keeping with this ventricular morphology concept. Specifically, we highlight results from histological studies, indicating that the appearance of cardiomyocytes can be different based on the sub-group of HLHS. In addition, we discuss the histological appearances of endocardial fibroelastosis (EFE), which is a common feature of one specific sub-group of HLHS. Lastly, we suggest investigations that should ideally be undertaken using HLHS myocardial tissues at early stages of HLHS development to identify biological pathways and aid the understanding of HLHS aetiology.

## 1. Introduction

Hypoplastic left heart syndrome (HLHS) is a collective term for a group of rare, severe cardiac malformations characterised by a specific combination of ventricular, valve and vascular abnormalities [1,2,3,4]. In all cases the morphological left ventricle is underdeveloped, such that the apex of the heart is represented by the right ventricle, both the mitral and aortic valves display variable atresia or stenosis, and the ascending aortic segment is diminutive. HLHS accounts for up to 3% of all congenital heart malformations and affects ~1 in 5–10,000 live births [5,6,7]. Although initially described by Lev as hypoplastic left heart complex [8], the term HLHS was later introduced in a clinical review that included pathologies such as coarctation of the aortic arch, that may also have reduction in left ventricular volume [9]. More recently, a nomenclature committee has defined HLHS and specifically excluded this and other malformations which, although may have ventricular hypoplasia, are dominated by another malformation with a distinctly different developmental origin, for example unbalanced atrioventricular septal defect, double outlet right ventricle and heterotaxy [1,3,10,11]. Despite this refinement in definition, the developmental failure and underlying causes of HLHS remain elusive.

## 2. Morphological Sub-Types of HLHS

**Figure 1:**
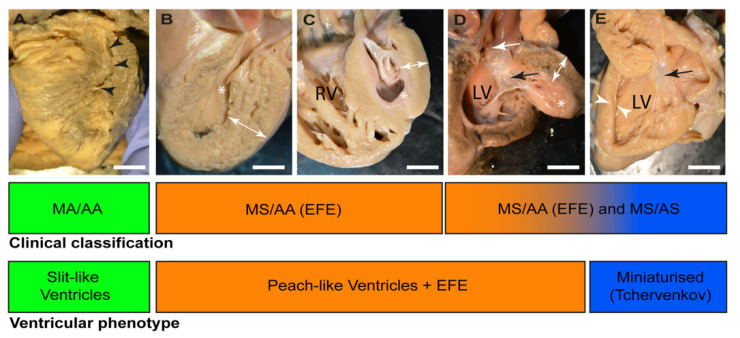
Clinical and morphological classifications of HLHS. (**A**) mitral atresia and aortic atresia (AA/MA). The posterior surface of the ventricular mass has been shaved to reveal a slit-like ventricular cavity phenotype (black arrow heads) without gross evidence of endocardial fibroelastosis (EFE). The apex of the heart is formed by the right ventricle. (**B**). Free left ventricular wall of a heart with mitral stenosis/aortic atresia (MS/AA). There is endocardial fibroelastosis seen on the inner surface of the left ventricular cavity (*) and the left ventricular wall is thickened (white double arrow). (**C**). Further example of MS/AA with thickened left ventricular wall (double white arrow) and prominent EFE (*) The right ventricle (RV) forms the apex of the heart. (**D**). HLHS heart with mitral stenosis/aortic stenosis (MS/AS) with the left ventricle (LV) opened to show prominent EFE (*) and thickened left ventricular wall. There is a stenotic aortic valve (white arrow), and the mural leaflet of the small mitral valve is also seen (black arrow). The appearances of the sectioned ventricle in (**B**–**D**) give the appearance of a peach that has been cut in half and the stone removed. (**E**). Heart with MS/AS with small left ventricle but with normal ventricular wall thickness (white arrow heads). The small mitral valve is also shown (black arrow). This heart with a miniaturised left ventricle has no EFE and contrasts with the examples of MS/AA and MS/AS with EFE in (**B**–**D**). Bar in (**A**–**E**) = 2 mm.

In clinical practice, sub-categories of HLHS can be described based on patency of the aortic and mitral valves. If the valve appears not to have an open orifice or no blood flow can be detected passing through, it is described as atretic; whilst if there is an apparent narrowing—either observed or inferred—it is described as stenotic. It is also possible to comprehensively re-categorise the valvar sub-types into three sub-types defined by left ventricular morphology [2]. Mitral atresia with aortic atresia (MA/AA) remains a sub-group common to both valve and ventricular morphological classifications, as the presence of mitral atresia with an intact inter-ventricular septum is always associated with a slit-like ventricle, found with difficulty in the posterior wall of the right ventricle. This sub-group showed a frequency of 36 to 46% of all HLHS cases [6]. The remaining HLHS hearts segregate differently between classifications. Some hearts previously described as examples of mitral stenosis and aortic stenosis (MS/AS), and showed a frequency of 23–26% of all HLHS cases [6]. Some of these hearts have miniaturised left-sided structures without the presence of endocardial fibroelastosis (EFE) or apparent thickening of the rudimentary left ventricle. These miniaturised hearts have been identified as a sub-set particularly suitable for biventricular surgical repair [12]. The remaining hearts with MS/AS have a common ventricular appearance to those hearts with mitral stenosis and aortic atresia (MS/AA), which had a prevalence of 20–29% of all HLHS cases [6]. Both groups of hearts with MS/AS and MS/AA exhibit EFE, and despite a small left ventricular cavity, do possess a thickened left ventricular wall. Together, they can be described as a peach-like ventricular group. It follows from this ventricular morphology-based re-classification that there should be common appearances between MS/AS and MS/AA hearts with EFE that are different to MS/AS with miniaturised features and MA/AA with slit-like ventricle (see Figure 1). The purpose of this review is to evaluate the published human-based studies of the myocardium in HLHS, asking if the available evidence is in keeping with this ventricular morphology concept and addressing whether there is any evidence pointing to a myocardial based aetiology for HLHS. Animal studies will only be briefly mentioned where there is specific information relating to the myocardium.

## 3. The Developmental Origins of the Normal Left Ventricular Myocardium

The slit-like ventricle appearances of MA/AA appear to be present from the earliest stages of heart development that can be visualised with ultrasound scanning and do not develop in later stages of pregnancy. In contrast, HLHS with peach-like ventricular morphology, that is MS/AS and MS/AA associated with endocardial fibroelastosis, have been shown to develop in either the second or third trimesters of human pregnancy when the human ventricular myocardium is already well formed [13,14], suggesting but not proving alternative aetiologies.

Animal studies have shown that the left ventricular (LV) myocardium and its endocardium are derived from cardiac progenitors that formed the initial heart tube (first heart field). This is in contrast to the right ventricle (RV), intraventricular septum and majority of atrial tissue, which are derived from the second heart field that add to the heart tube at later stages [15]. The initial left ventricular chamber has a thin wall which then thickens before the trabeculae, finger-like projections of myocardium, extend into the cavity at Carnegie stage 12 when the human embryo is 26 days old. Myocardial thickening continues over the next week and at around 33 days of development (Carnegie stage 13) ventricular septation takes place [16]. The large surface area of the trabeculae is initially important in oxygen delivery to the myocardium, but as development proceeds, oxygen delivery to the cardiac muscle via the surface of the trabeculae becomes limiting and developing coronary vessels take over this role [16]. As proliferation of the trabecular and compact layer cardiomyocytes progress, there is mixing of these cells in a middle hybrid layer as demonstrated in mouse studies [17]. Animal studies have shown the importance of the epicardium in providing trophic signals for growth of the compact layer. In contrast, the growth of trabeculae is dependent on endothelial signalling through the Notch pathway and neuregulin [16]. However, by approximately 9 weeks of development—well before HLHS is first recognized—there is remodelling of the surface trabeculae resulting in a smooth left ventricular surface with only residual fine trabeculae at the ventricular apex [16].

## 4. The Myocardial Architecture in HLHS

**Figure 2:**
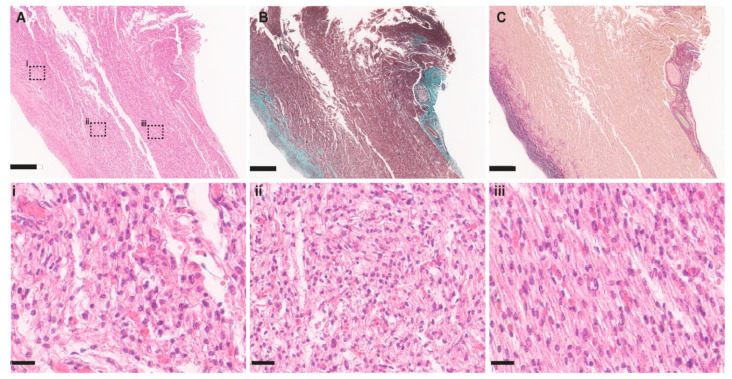
Histological appearances of HLHS heart with peach-like ventricular phenotype (MS/AS and EFE). (**A**–**C**) Sections through free wall of left ventricle. (**A**) H&E stain of left ventricular free wall. (**i**) High powered views of the myocardium show marked cardiomyocyte disarray and vacuolated cardiomyocytes in areas close to the endothelial surface. (**ii**) Further way from the lumen there is evidence of myocardial disarray, but the vacuolated cardiomyocyte appearances are less prominent. (**iii**) The epicardial side the myocardium appears ordered and the cardiomyocytes grossly normal. (**B**) Masson’s trichrome stain showing collagen deposits (blue) in the sub-luminal myocardial wall. This affects the areas of myocardium with most cardiomyocyte disarray. (**i**) Millers’ elastin stain shows prominent elastin fibre deposition black staining) in luminal part of the left ventricular myocardium. Scale bar in (**A**–**C**) = 500 um, in (**i**–**iii**) = 200 um.

Macroscopically, the LV myocardium of slit-like MA/AA HLHS hearts is very different from that seen in those hearts with peach-like MS/AA or MS/AS. In the slit-like MA/AA HLHS hearts, the LV is included within what is considered to be the posterior wall of the RV. Since this posterior wall is the same thickness as the rest of the RV wall yet encompasses both the LV free wall and the interventricular septum, it follows that both these structures are thinner than normal. In contrast, although the ventricular chamber is small in peach-like hearts, there is variable but marked thickening of the ventricular wall with obvious endocardial fibroelastosis, which gives the appearance of a peach that has been cut in half (Figure 2). Although the appearance of cardiomyocytes within the thinned LV walls of the slit-like hearts are described as being normal [18,19]. It is important to emphasise the almost complete lack of published studies that relate directly to the slit-like ventricular (MA/AA) phenotype. Neither the cellular components nor the extracellular matrix appearances have been clearly described. Indeed, the studies referenced in this review essentially focussed on the peach-like hearts with EFE (with MS/AA or MS/AS), although, again, there have been no studies which clearly indicated whether the apparent wall thickening of the hypoplastic LV in these hearts is due to either cardiomyocyte hypertrophy, cardiomyocyte hyperplasia, increased extracellular matrix or any combination of these.

## 5. Cardiomyocyte Organisation

Each cardiomyocyte is enveloped in a connective tissue sheath, the endomysium, and several cells are further organised into fibres by thicker areas of connective tissue, the perimysial sheaths. Within this scaffold, the cardiomyocytes remain as discrete cells but connect and communicate with each other at their ends, through intercalated discs, creating an ordered array of contractile components. Further orientation of fibres into sheets within the myocardial wall is evident orientations [16]. In the formed mature heart, the right ventricle tends to wrap around the left ventricle, but both ventricles have walls that are macroscopically organised into layers of fibres with oblique orientations [16]. Longitudinal running muscle layers are found at the inner and outer parts, and in between them are helically orientated fibres. Although there are some apparently disorganised cardiomyocytes at the apical end of the interventricular septum where the left and right ventricular myocardium meet, in the normal heart there is little evidence of any cardiomyocyte disorganisation. It is not known to what extent this fibre orientation is based on a genetically-regulated developmental process or is secondary to mechanical forces. One of the most obvious abnormalities in hearts with peach-like thickening and EFE, i.e., MS/AS or MS/AA, is myocardial disarray affecting the inner two thirds of the left ventricular wall [18,19]. Although myocardial disarray was initially thought to be pathognomonic and specific for hypertrophic cardiomyopathy, it is also found in congenital heart malformations where the ventricles have abnormal wall loading due to obstructed outlets, for example pulmonary atresia [20]. Although most HLHS studies do not report disarray in the right ventricle [18,19], one histological study of HLHS myocardium, examining hearts aged from 6 postnatal days to 6 months, did indicate such changes also affecting the right ventricle [21]. It is likely that this right ventricular abnormality may be also secondary to changes in right ventricular loading in failing hearts. There is a need for these issues to be addressed in well-designed comprehensive studies.

## 6. Cardiomyocyte Ultrastructure in the Slit-like and Peach-like Ventricles

In addition to disorganised arrangement of cells, the ultrastructural organisation of the cardiomyocytes is also grossly disrupted in the peach-like MS/AS; MS/AA hearts. Histological studies indicate the cells are vacuolated, have reduced cytoplasm and are of variable size [21]. However, these studies have universally been carried out in late gestation and/or postnatal HLHS cases and thus may not be representative of the early stages of pathology. In similar studies performed by the Toronto group, the nuclei have been described as being ovoid and centrally placed, presumably implying these are newly replicated cardiomyocytes. Similarly, chromatin deposits implying DNA remaining after cell death have also been reported [22]. Again, these studies have examined myocardium late in the evolution of this HLHS phenotype and it is not known whether these are late changes of a profoundly damaged and potentially scarred myocardium or whether they represent a primary aetiology.

An important aspect of myocardial biology is the close alignment of mitochondria to sarcomeres, thought to be essential to meet the high energetic requirements of the tissue. There is a paucity of electron microscopy data, required to visualise the cardiac mitochondria for HLHS cases. The images that do show these organelles also indicate a very abnormal sclerotic myocardium and are not sufficient to determine relationships to sarcomeres [22].

It has been suggested from immunohistological data, that Connexin 43, the gap junction protein responsible for electrical coupling of ventricular cardiomyocytes, is absent in the peach-like MS/AS; MS/AA hearts [23,24]. However, this finding has not been confirmed and may represent a late change in severely disturbed cardiomyocytes. Similarly, it has been suggested that PECAM1, a surface antigen that is considered highly specific for endothelial cells, is aberrantly expressed by cardiomyocytes in these hearts [21]. This has not been confirmed and a recent study used PECAM1 antibody to specifically identify endothelial cells from cardiomyocytes by FACS [22], suggesting that endothelial cells express PECAM1 and other cells, i.e., cardiomyocytes, do not.

## 7. Endocardial Fibroelastosis in Peach-like Ventricles

The cardinal feature of the peach-like HLHS hearts with either MS/AS or MS/AA is the presence of EFE. When established, EFE is recognised visually as an opalescent, white coating that extends over the mitral and aortic valve surfaces. On echocardiography, EFE presents as a highly echogenic signal on the inner myocardium and valves [25]. Histologically, EFE is recognised as an abundance of elastin fibres, collagen and smooth muscle cells expanding the potential space between the endothelium and myocardium, but a predominantly fibrotic infiltrate also extends into the inner part of the myocardium [26].

EFE is recognised in a wide range of situations that have dilation of the ventricle as a common finding, especially dilated cardiomyopathy [26]. It has historically been associated with viral infections and viral particles have been described within it [26,27,28]. The reduction in childhood infections, such as mumps, in recent years, has been suggested as a reason why EFE in childhood has become less common [26]. Although EFE appears in certain pathological conditions at all ages, it is much more exuberant prior to birth, predominantly occurring in young infants, showing an inverse correlation with age [29]. With respect to HLHS, there have been few studies, but the EFE appearances seem similar to examples of isolated EFE or that found in association with childhood- or adult-dilated cardiomyopathy, where much more is known [13,30]. 

The histological appearances of EFE are well recognised. The sub-endocardial space normally contains very little connective tissue but does contain occasional cells with the appearance of smooth muscle cells (SMCs). As EFE develops, there is an increase in the number of these cells, which are the likely source of collagen and elastin. Electron microscopy shows that they contain electron dense cytoplasm, myofilaments and vesicles, which is in keeping with the phenotype of SMCs [26]. Immunohistochemical analyses have supported that these cells possess a SMC phenotype as they express the same markers as the vascular SMC of the coronary circulation, including alpha smooth muscle actin, smooth muscle myosin, desmin and calponin. In contrast, the cells do not immunolabel with fibroblast markers, such as vimentin, fibronectin, periostin or tenascin [31]. The source of these cells is presumed to be the underlying endothelium through endothelial to mesenchymal transition (EndoMT). In support of this, cells in this compartment have been shown to co-label with antibodies against an endothelial-specific antigen, CD31 and alpha smooth muscle actin [32]. The endothelium in HLHS also expresses markers of EndoMT such as SLUG, SNAIL and TWIST, which also support the hypothesis that the SMCs are derived from these endothelial cells [33]. Electron microscopy suggests that cells further away from the sub-endothelial space are more fibroblastic in phenotype [26] and although it is suggested that the SMC-like cells within the sub-endothelial space give rise to these cells, there is no solid evidence to support this. Indeed, there are abundant fibroblasts in this region of the outer ventricular wall that could participate in this fibrotic reaction. Limited studies have been carried out in HLHS samples. A study of well-established EFE from (peach-like) MS/AS; MS/AA late foetal hearts showed an increase in collagen I immunolabelling [22]. The examination of resected EFE showed collagen and elastin through both histological and immunohistochemical methods and indicated active elastase and gelatinase activity through in situ zymography. However, there were no statistical differences between patients across a range of ages up to 5 years, thus it was not possible to exclude the failure of matrix turnover as a reason for EFE accumulation [32]. Whilst this study was carried out in samples removed from children with critical aortic stenosis and EFE, rather that HLHS, it is likely to be relevant as many of these patients will have also had reduced left ventricular cavity size.

Transgenic Cre-lox-based lineage tracing studies in mice have provided some insight as to the origins of the cells involved in EFE and both support and extend the human studies. Whilst an in-depth discussion of these models is beyond the scope of this human pathology review, it is useful to note parallels. The experiments are based on heterotopic heart transplantation, where a heart is surgically implanted into the vascular system of another animal. Experiments show that EFE only develops if transplanted mouse hearts are less than 2 weeks of age [34,35] and if they have been transplanted in a configuration that introduces the unloading of ventricular strain. Experiments using the Tie2-Cre transgenic construct, which allows labelling of all endothelial cells and their derivatives, have shown that cells in the sub-endocardial space originally derive from endothelial cells and that this is by EndoMT [33]. Further Cre-lox lineage-tracing experiments indicate that the majority of fibroblasts that proliferate in the adjacent myocardium are part of the complement of fibroblasts that are originally derived from the epicardium [36]. In this regard, there is the usual difficulty in identifying fibroblasts specifically from other cells. For example, FSP1 is not only expressed by fibroblasts but also endothelial cells, smooth muscle cells and myeloid cells [37].

## 8. Histological Evidence of Myocardial Conditions Leading to HLHS

The initial insults that lead to the thickened and EFE-lined ventricles of the peach-like MS/AS; MS/AA or the slit-like ventricle morphology of MA/AA remain unknown. Whilst it is recognised that a primary cardiomyopathy can provoke EFE, or a cardiopathy-related genetic variant could affect outcome in HLHS [38,39], the evidence for this causing HLHS in human patients does not exist and the myocyte disarray observed is likely to be a secondary consequence of an earlier primary pathology. Similarly, inflammatory conditions, such as auto-immune phenomena or viral infections, have been suggested. However, there is no evidence that maternal-derived antibodies could lead to HLHS phenotypes in the same way as they can cause congenital heart block [40,41]. EFE has been associated with viral infections and viral particles have been described within the EFE layer [27,28]. However, despite the possibility of a minor increase in the incidence of HLHS in the spring [42] or summer months [43], there is no direct evidence that viral infections lead to HLHS. Studies have not been carried out to identify a T cell or macrophage infiltrate that would provide important evidence to support or disprove these aetiologies.

Tissue hypoxia, as indicated by the elevated expression of HIF1A and Vegf, has been described in the myocardium of HLHS hearts with EFE in the context of MS/AS and MS/AA. There was evidence of cardiomyocyte senescence and DNA damage through the increased expression of H2Ax but without evidence of shortened telomeres [22]. Taken together, this might suggest that myocardial hypoxia is an important process in the progression of HLHS. However, these studies were performed in late gestation hearts with severe EFE, profound myocardial disarray and extensive fibrosis. Thus, these markers may not indicate a primary hypoxic event but instead be representative of a secondary proliferative/fibrotic response or perhaps secondary hypoxia due to the fibrotic EFE barrier. For example, the expression of HIF1α is associated with a normal mid-gestational proliferative phase in hypoxic cardiomyocytes [44]. Thus, its upregulation might be a compensatory process in response to a reduced initial complement of cardiomyocytes or be a component of a hyperplastic response to loss of cardiomyocytes or poor myocardial function. Of particular importance is the evidence of DNA damage yet preserved telomere length in HLHS hearts [22]. This might appear to exclude proliferative exhaustion as a cause for the senescent phenotype as the telomeres are not shortened, but as there is expression of telomerase in human embryonic tissues and foetal hearts [45], telomere length may be maintained despite cardiomyocyte proliferation and progressive DNA damage. The implication of high levels of senescent cardiomyocytes in HLHS hearts is also unclear. Much is known about senescence in aging and the potential benefits of clearing senescent cells from aged hearts. However, it is possible that senescence may play a positive role during development and/or regeneration. For example, the senescent phenotype has been associated with reactivating developmental pathways, aiding regeneration in the heart, promoting angiogenesis and limiting fibrosis [46].

## 9. Vascular Abnormalities in HLHS

In the developed heart a rich network of endothelial-lined channels provides the dense myocardium with nutrients and oxygen. These capillaries lie alongside the bundles of cardiac muscle fibres and connect with the epicardial vessels that branch from the main coronary arteries on the surface of the heart. Distally they connect to the cardiac veins and drain into the right atrium through the coronary sinus. However, there are also connections between this coronary circulation and the chambers of the heart. Although there has been a relaxed usage of nomenclature, it is appropriate to describe the veno-luminal vessels that connect to the venous side of the cardiac capillary bed as Thebesian veins, whilst the arterio-luminal vessels connecting chambers to the arterial side of the capillary bed as vessels of Wearn [47]. In addition, there are sinusoidal arterial channels on the arterial side of the coronary circulation that connect to the chambers of the heart, which are irregular in outline and are composed only of endothelial cells without vascular smooth muscle support [47,48]. The patterns of the coronary arteries in HLHS have been well described, although they are largely confined to minor positioning defects where the left or right coronary artery is not completely centred in the sinus [49]. The anterior descending, or interventricular, artery is an important and consistent landmark for identifying the extent of the slit-like ventricle in MA/AA. The overall dominance pattern of the coronary circulation is indicated by which main coronary vessel supplies the posterior interventricular artery and normally this is the right coronary artery. Left dominance or a right/left balanced dominance occurs in the majority of peach-like hearts with EFE, with left dominance also seen in approximately half the hearts with a slit-like ventricle [50]. Both the reason for this and its implications are unclear but may represent coronary blood flow that is unable to enter the left ventricle, passing on to the posterior interventricular artery. This aberrant blood flow pattern may explain why coronary artery tortuosity and mural thickening is limited to the vessels on the LV wall in MS/AS and MS/AA [19]. A major concern for some clinicians has been the prevalence of atypical connections between the coronary vessels and the chambers, mainly a feature of MS/AA hearts. Sinusoidal channels have commonly been seen on histological sectioning [51]; however, connections to the arterial side of the coronary circulation have also been noted [19,52] but importantly only found in cases of MS/AA and not in MA/AA. The implications of these connections are unclear as they are more frequently found post-surgery [52] and are usually small [53]. Thus, adverse outcomes following surgery may have been more related to technical issues during surgery rather than myocardial ischaemia resulting directly from these coronary vascular connections [54].

Lymphatic channels are also present, draining extracellular fluid and circulating cells, but there is no clinical evidence of specific abnormalities in the pre-surgical HLHS heart, although problems are related to palliative operations such as bi-directional Glenn and Fontan anastomoses [55].

## 10. Conclusions and Perspective

**Figure 3:**
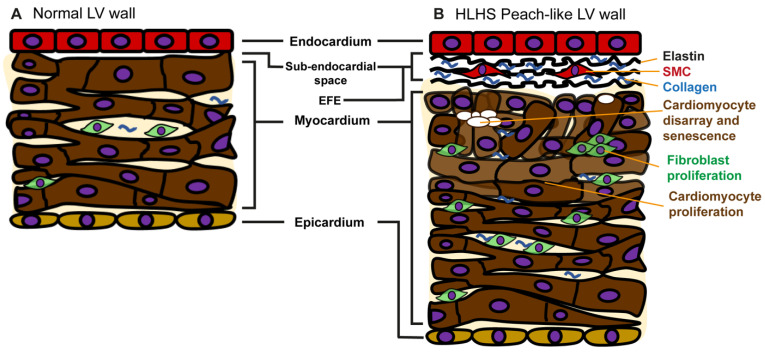
Cartoon showing changes found in HLHS peach-like left ventricular wall. (**A**). In the normal left ventricular (LV) wall the sub-endocardial space is not prominent, and cardiomyocytes are organised in parallel fibres. Between the cardiomyocytes there are fibroblasts (**green**) and there is also diffuse collagen deposition (**blue**). (**B**). In the peach-like LV wall seen in hearts with both MS/AA and MS/AS, the sub-endothelial space is enlarged and there are prominent elastin fibres (**black**) with increased collagen deposition (**blue**). There are also smooth muscle cells (SMC; **red**), which are considered to derive from the endothelium. The myocardium close to the lumen has marked disarray and there are vacuolated cells. There is more collagen deposition in this area. Towards the epicardial side of the LV wall the cardiomyocytes appear more normal and normally organised. Overall, the LV wall appears thickened, and this may be due to cardiomyocyte hyperplasia, fibroblast proliferation or more extracellular matrix deposition or a combination of these changes.

This evaluation of human-based research indicates that remarkably little has been done so far to understand the myocardial substrate in HLHS, particularly in the examples of MA/AA with the slit-like ventricular morphology. Moreover, studies on myocardium in the setting of EFE in hearts with MS/AS or MS/AA have involved very late stages in the natural history of the disease when any primary pathologies have been potentially overwhelmed by severe sclerotic changes and cardiomyocyte loss (Figure 3). In large part, this is due to the difficulty in obtaining appropriate samples from the LV, and its associated valves and structures from these early stages. Whilst some of this lack of material is due to the improvements in surgical intervention making post-natal survival more possible, it is clear that the termination of pregnancy does not generally lead to samples being taken for research-orientated analysis. Many days can pass between the cessation of the pregnancy and post-mortem examination with the inevitable autolysis of tissues. The answer to this lies with foetal medicine departments and perinatal pathologists being persuaded to obtain tissues rapidly, carry out routine stains or immunolabelling and preserve tissues for RNA and DNA analysis. Whilst high-quality clinical imaging is now routine, the detailed recording of the valve and ventricular morphology is essential to allow effective analysis as our understanding of the condition advances, and paediatric and foetal cardiologists must record the anatomy as well as their interpretation. Another issue is the persistence of information within the accepted literature that has not been validated or is probably incorrect. For example, PECAM1 overexpression in cardiomyocytes has been suggested [21] and obviously refuted through FACS analysis [22]. The relevance of several aetiological ideas that remain within the literature is unclear and these could be excluded as major factors through modern analyses of the myocardium. For example, infective or inflammatory conditions are suggested to be involved in HLHS and markers for macrophages and T cells should be applied to LV tissue samples. Finally, recent findings suggesting the activation of senescence pathways, HIF1α and VEGF [22] are important and should be confirmed independently. They also need to be evaluated at much earlier stages of HLHS development. Similarly, in-depth histological analysis of myocardium in the heterotopic transplant model and in resected EFE from critical AS hearts should be performed to understand the relevance of these models to the HLHS process.

## Data Availability

The human data that support the findings of this study are openly available in [2] at doi: 10.1186/s13023-017-0683-4, and are available from the corresponding author on reasonable request.
